# Relationship between Body mass index (BMI) and body fat percentage, estimated by bioelectrical impedance, in a group of Sri Lankan adults: a cross sectional study

**DOI:** 10.1186/1471-2458-13-797

**Published:** 2013-09-03

**Authors:** Chathuranga Ranasinghe, Prasanna Gamage, Prasad Katulanda, Nalinda Andraweera, Sithira Thilakarathne, Praveen Tharanga

**Affiliations:** 1 Allied Health Sciences Unit, Faculty of Medicine, University of Colombo, PO box 25, Kynsey road, Colombo 10, Sri Lanka; 2 Diabetes Research Unit, Faculty of Medicine, University of Colombo, PO box 25, Kynsey road, Colombo 10, Sri Lanka

**Keywords:** Body mass index, Body fat, Bioelectrical impedance, Sri Lanka, Adults, Age, Sex

## Abstract

**Background:**

Body Mass Index (BMI) is used as a useful population-level measure of overweight and obesity. It is used as the same for both sexes and for all ages of adults. The relationship between BMI and body fat percentage (BF %) has been studied in various ethnic groups to estimate the capacity of BMI to predict adiposity. We aimed to study the BMI–BF% relationship, in a group of South Asian adults who have a different body composition compared to presently studied ethnic groups. We examined the influence of age, gender in this relationship and assessed its’ linearity or curvilinearity.

**Methods:**

A cross sectional study was conducted, where adults of 18–83 years were grouped into young (18–39 years) middle aged (40–59 years) and elderly (>60 years). BF% was estimated from bioelectrical impedance analysis. Pearsons’ correlation coefficient(*r*) was calculated to see the relationship between BMI-BF% in the different age groups. Multiple regression analysis was performed to determine the effect of age and gender in the relationship and polynomial regression was carried out to see its’ linearity. The relationships between age-BMI, age-BF % were separately assessed.

**Results:**

Out of 1114 participants, 49.1% were males. The study sample represented a wide range of BMI values (14.8-41.1 kg/m^2^,Mean 23.8 ± 4.2 kg/m^2^). A significant positive correlation was observed between BMI-BF%, in males (*r* =0.75, p < 0.01; SEE = 4.17) and in females (*r* = 0.82, p < 0.01; SEE = 3.54) of all ages. Effect of age and gender in the BMI-BF% relationship was significant (p < 0.001); with more effect from gender. Regression line found to be curvilinear in nature at higher BMI values where females (p < 0.000) having a better fit of the curve compared to males (p < 0.05). In both genders, with increase of age, BMI seemed to increase in curvilinear fashion, whereas BF% increased in a linear fashion.

**Conclusions:**

BMI strongly correlate with BF % estimated by bioelectrical impedance, in this sub population of South Asian adults. This relationship was curvilinear in nature and was significantly influenced by age and gender. Our findings support the importance of taking age and gender in to consideration when using BMI to predict body fat percentage/obesity, in a population.

## Background

Overweight and obesity are defined as abnormal or excessive fat accumulation in the body that may impair health [1]. During the last few decades, the prevalence of obesity has increased and has become a considerable global health hazard [2,3]. Excessive body fat is associated with increased metabolic risk, and its’ measurement is important in implementing curative and preventive health measures. Direct measurement of body fat requires sophisticated equipment and is time consuming, it is also difficult in epidemiological studies [4]. The most commonly used surrogate measure for prediction of body fat percentage (BF%) is Body Mass Index (BMI) [5-8]. How ever some studies have shown, that they (BMI-BF%) have an imperfect association [9] and some in contrast justify a strong association [5,6,9-12]. Some even have described the linear [6] or curvilinear [8,9] nature of this relationship.

World Health Organisation (WHO) also recommends BMI as the most useful population level measure of overweight and obesity, and is used as the same for both sexes and in all ages of adults [1]. So BMI of >25 kg/m^2^ and >30 kg/m^2^ are considered to be overweight and obese in adults irrespective of gender and age. This use of a single standard for obesity for all adults was recommended because it is thought to be independent of age and it can be used for making comparisons across studies [13]. At present, there are studies conducted in various ethnic groups to determine the effect of age and gender [14,15] in the BMI -BF% relationship, and there are uncertainties about the final conclusion [9]. Published data on this topic is limited in South Asians, who have relatively high BF% and increased cardiovascular risk compared to other ethnic groups [16].

We used Bioelectrical Impedance Analysis (BIA) method to estimate the Body fat %. BIA is known to provide a rapid, non-invasive and relatively accurate measurement of body composition [17] with the possibility of utilizing at field settings. BIA methods validity has been tested, taking BF% as the outcome variable; with a range of reference techniques including, total body water hydrodensitormetry, dual energy X-ray absorptiometry and air displacement plethysmography [9,18]. Large population studies conducted even have provided reference values of body composition based on bioelectrical impedance analysis [19].

So we studied a large sub-population of South Asian adults from Sri Lanka; to determine the relationship between BMI and BF %, and then to identify the nature of the relationship, whether linear or curvilinear. We also tried to find the effects of age and gender on this relationship. We wish to present our results which would add more evidence to the ongoing discussion; as they were derived from an ethnic group which was not studied before.

## Methods

### Study population

The study was designed as a cross-sectional (population) study. The participants were randomly recruited (every 3rd person who volunteered) from those attending a research center located in an exhibition venue during a medical exhibition conducted by, Faculty of Medicine, University of Colombo Sri Lanka in 2008. Adults above the age of 18 years with no other physical disease were included. Pregnant women were excluded. Height, weight and body composition measurements were carried out by a group of medical graduates after supervised training. Inter-observer/operator reliability was assured. Informed written consent was taken from all participants. Confidentiality was maintained during the storage, retrieval and analysis of data. Ethical approval was taken by Ethics Review Committee Faculty of Medicine University of Colombo Sri Lanka.

### Body composition measurements

#### ***Anthropometry***

Measurements were taken using standardized equipment. Height of all participants were measured using a stadiometer *(seca 206,* Germany) in standing position without footwear to the nearest 0.1 cm. Weight was measured with minimum clothes using a calibrated electronic scale with digital readout (*seca 808*, Germany) to the nearest 0.1 kg. BMI was calculated by weight (kg) divided by height (m) squared (kg/m^2^).

#### ***BIA derived percent body fat***

Total body fat percentage (BF %) was estimated by using a commercially available single-frequency, 8 electrode bio impedance analyzer system (BC-418, Tanita Corp, Tokyo, Japan). The reliability and validity of this system in measuring BF% has been previously verified in multiple ethnicities [20,21]. All measurements were taken during morning hours (0830–1200) and the subjects didn’t have any vigorous activity during the preceding 12 hours of the measurement. The system consisted of two handgrips with two electrodes each and a footplate with four electrodes. All procedures carried out according to manufacturer instructions [20]. The electrodes between the left and right grips were short-circuited, along with those for the left and right feet. Study subjects stood on the footplate and gently grasped the two handgrips with arms held straight forward at 90 degrees. During the measurement, the instrument recorded whole body impedance from the hands to the feet by applying an electric alternating current flux of 0.8 mA at an operating frequency of 50 kHz. Finally, BF% was calculated from the whole body impedance value and the pre-entered personal data (age, gender, height and weight) of the corresponding subject. BF% was estimated to the nearest 0.1%. Inter-observer /operator reliability and precision of impedance measurements in the same subjects under standard condition were monitored. Whole-body composition was estimated using standard equations provided by the BIA manufacturer.

### Statistical analysis

Subjects were grouped into males and females. Then each gender was grouped as young (18–39 years) middle age (40–59 years) and elderly (>60 years). Basic descriptive statistics for subject data were expressed as means ± standard deviations. Differences between means were separated by one way ANOVA.

Pearsons’ correlation coefficients (*r*) were calculated to assess the link and the degree of relation between BMI and BF%, in relation to gender and age variables. Multiple regression analysis was performed to examine the possible effect of gender on the relationship between BMI and BF%. Then age was further added to the model to see its’ effect. BMI, age and gender were taken as independent variables and BF% as the dependent variable.

Polynomial regression analysis examined the linearity of the BMI-BF% relationship. General linear model analysis was first used. Then it was extended to examine non-linearity by including a quadratic term for BMI (BMI^2^). Variance of BF% was estimated for general linear modal; and after adding the quadratic term. This was performed in males and females separately. Visual inspection of the relationship (BMI-BF %) was also made. Distribution and linearity of age-BMI and age-BF% relationships were separately assessed. Statistical analysis of data was carried out using the SPSS version 16.0 (SPSS Inc. USA) software for Windows.

## Results

### Baseline group characteristics

A total of 1114 adults were investigated during the study; 49.1% were males (Table 1). The study sample represented a wide range of BMI values (14.8 - 41.1 kg/m^2^). Ninety four percent (94%) of the total sample had BMI values <30 kg/m^2^ (Mean 23.8 ± 4.2 kg/m^2^).

**Table 1:** Male and female characteristics (mean ± SD) contrasted by age group

**Variable**	**Male (n = 547)**			**Female (n = 567)**		
	**Young**	**Middle-age**	**Elderly**	**Young**	**Middle-age**	**Elderly**
	**(n = 279)**	**(n = 232)**	**(n = 36)**	**(n = 260)**	**(n = 279)**	**(n = 28)**
Age (y)	26.7 ± 6.6	48.4 ± 5.3	65.9 ± 6.5	25.9 ± 7.2	47.3 ± 4.9	64.6 ± 5.0
Height (cm)	168.7 ± 6.2	165.9 ± 6.0	162.2 ± 5.5	156.5 ± 5.8	155.1 ± 5.6	152.1 ± 6.8
Weight (kg)	64.9 ± 12.1	68.2 ± 11.4	60.5 ± 11.9	53.6 ± 11.4	62.4 ± 10.1	57.4 ± 24.7
BMI(kg/m^2^)	22.7 ± 3.8	24.6 ±3.5	22.8 ±3.9	21.8 ±4.3	26.1 ± 3.8	24.7 ± 3.5
Body fat%	19.5 ±6.6	24.5 ±4.6	24.4 ± 5.5	28.0 ± 6.0	34.7 ± 4.3	36.7 ± 4.8

Continuous variables (Age, Height, Weight, BMI-Body Mass Index, Body Fat%) are given as mean values with their standard deviations (SD). Number of participants (n).

### Relationship between BMI and BF%

There was a strong and significant positive correlation between BMI- BF% in males (*r* = 0.75,p < 0.01;SEE = 4.17) and in females (*r* =0.82, p < 0.01;SEE = 3.54). Correlations calculated for the three different age groups separately, also showed the significance (p < 0.01).In males/females they were, *r* =0.79/0.84 (young), *r* =0.71/0.70 (middle age), *r* =0.59/0.075 (elderly) respectively.

### The effect of age and gender in the BMI -BF% relationship

Age and gender were found to be significant predictor variables in the regression models (p < 0.000) (Tables 2 and 3), where gender contributing more effect to the relationship (Model 2).

**Table 2:** Multiple regression analysis for change in BF% with BMI, age for males and females (Model 1)

	**Male**			**Female**		
	**Intercept/Regression coefficients/R**^**2**^	**SE**	**Beta**	**Intercept/Regression coefficients/R**^**2**^	**SE**	**Beta**
Intercept	-9.662	1.011		-3.819	0.688	
BMI	1.114	0.043	0.678 (p < 0.000)	0.918	0.032	0.670 (p < 0.000)
Age	0.139	0.012	0.313 (p < 0.000)	0.153	0.011	0.331 (p < 0.000)
R^2^	0.654			0.764		

*SE*: Standard Error, *BMI*: Body mass index, *R*^*2*^: squared regression coefficients.

**Table 3:** Multiple regression analysis for change in BF% with BMI, age and gender (Model 2)

	**Intercept/Regression coefficients/R**^**2**^	**SE**	**Beta**	**p**
Intercept	-16.563	0.651		<0.000
BMI	1.004	0.026	0.532	<0.000
Age	0.143	0.008	0.248	<0.000
Gender	-9.343	0.204	0.586	<0.000
R^2^	0.818			<0.000

*SE*: Standard Error, *BMI*: Body mass index, *R*^*2*^: squared regression coefficient.

### Linearity/curvilinearity of the BMI -BF% relationship

**Figure 1:**
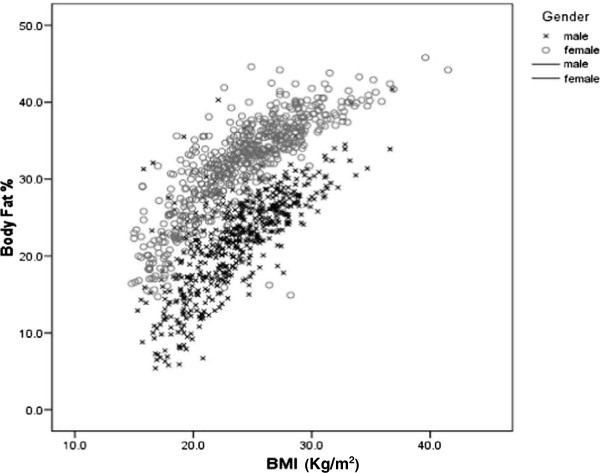
**Scatter plot of the relationship between Body Mass Index (BMI) and percentage of body fat (BF%) of Sri Lankan men (+) and women (o).** Relationship between the percentage of body fat (BF %) and body mass index (BMI) of Sri Lankan (+) males and (o) females. The linear regression models: (BF% male = (BMI × 1.114) + (age × 0.139) – 9.662 and BF% female = (BMI × 0.918) + (age × 0.153) +3.819.Polynomial regression for non linearity: females (R^2^ = 0.70, SEE 3.4%, p < 0.000) males (R^2^ = 0.58, SEE 4.1%, p < 0.05).

Visual inspection of the scatter plot (Figure 1) also showed the positive relationships between the BF % and BMI. It revealed that the relationship appears to be linear in nature and curvilinearity developing towards the high BMI values. Polynomial regression which was carried out to test for linearity in both males and females showed a significant quadratic component. The BMI linear component accounted for 67.5% of the female variance and 57.6% of the male variance. Adding the quadratic component accounted for an additional 2.9% of the female variance (p < 0.000) and 2.2% of the male variance (p < 0.01). The female model (R^2^ = 0.70,SEE 3.4%) provided more accurate fit than the male model (R^2^ = 0.58, SEE 4.1%). This confirmed that the relationship between BMI-BF% measured by bioelectrical impedance for this Sri Lankan group of adults was curvilinear.

### Independent relationship of age on BMI and BF%

**Figure 2:**
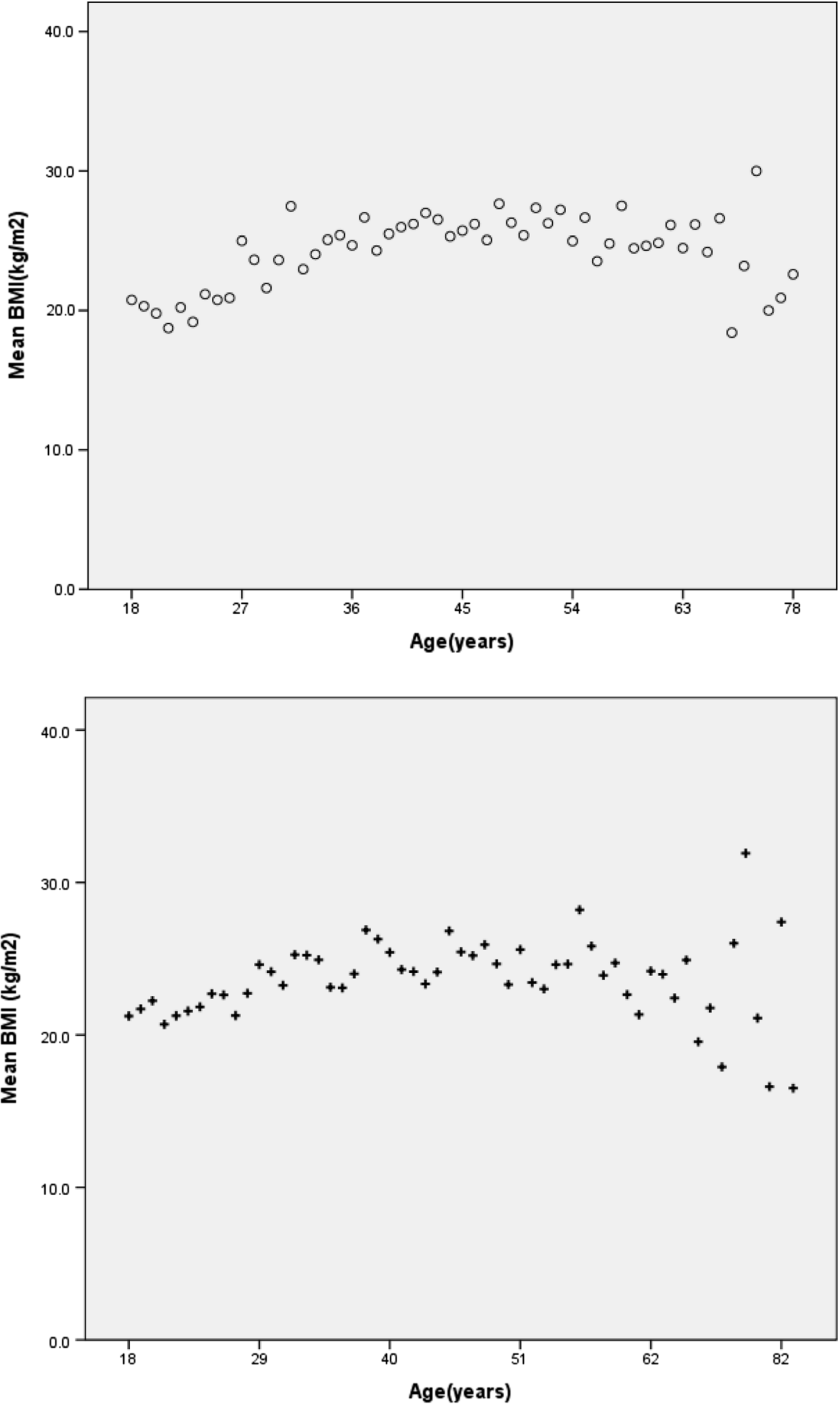
Relationship between BMI and age in (o) females (upper graph) and (+) males (lower graph).

BMI noted to increase with age in young; relatively constant in middle age and decline in elderly in both males and females (Figure 2). This curvilinear effect was more significant in females (R^2^ = 0.27, R^2^ change = 0.058, SEE 11.5%) (p = <0.000) compared to males (R^2^ = 0.61, R^2^ change = 0.009 ,SEE 13.7%) (p = <0.05). Females had a significantly higher mean BMI values than males in all three age group categories except in young (age 18–39 years) (p < 0.05) (Table 1).

**Figure 3:**
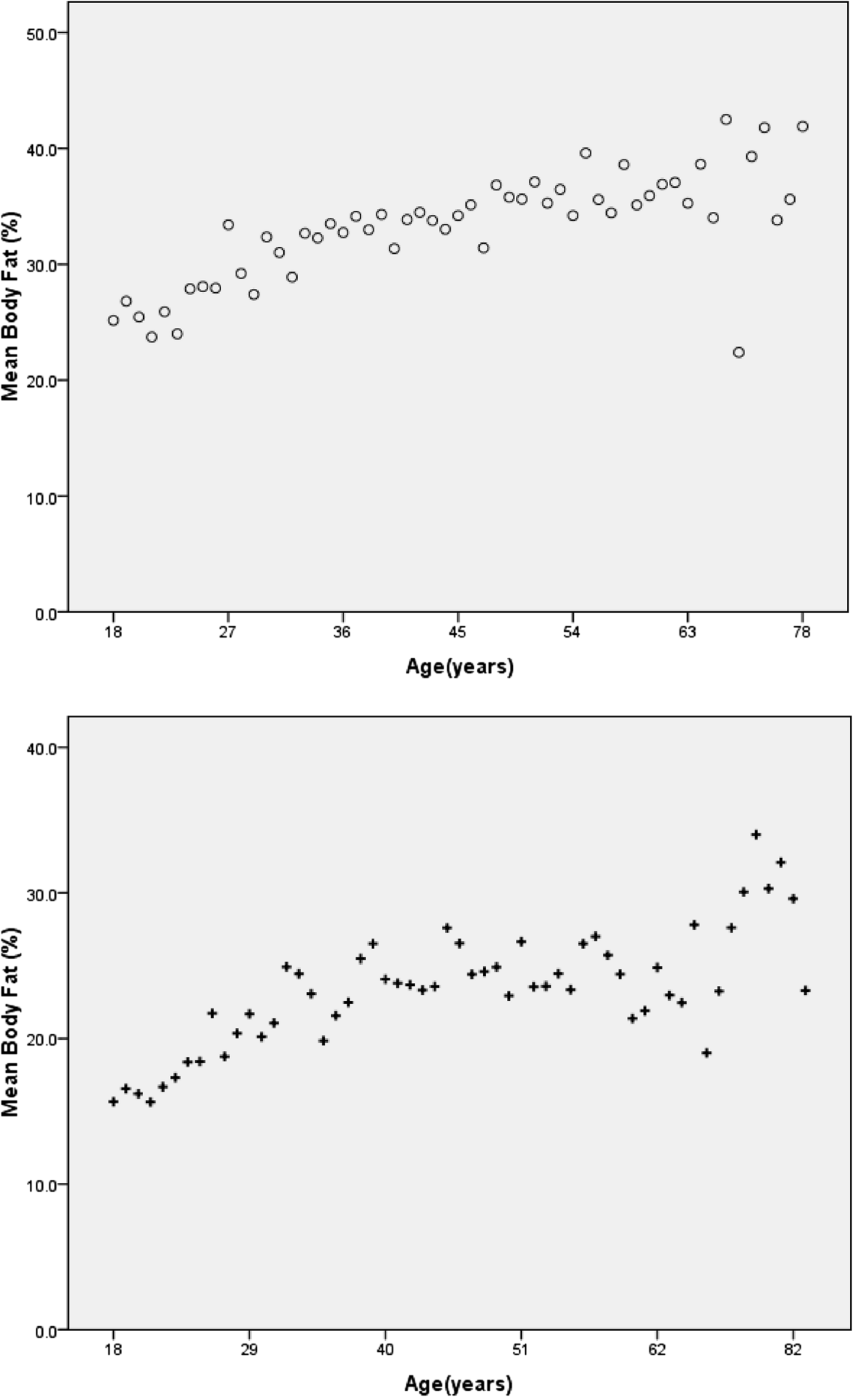
Relationship between percent body fat (BF%) and age in (o)females (upper graph) and (+)males (lower graph).

In both males and females BF% showed an increase with age (Figure 3) with a positive linear correlation (males *r* =0.47, females *r* =0.64; p < 0.000). Females of all ages had significantly higher total body fat than males (p < 0.001) (Table 1). The mean difference in BF% between females and males was 10.44.This difference was shown to increase with age (young 8.5, middle-age 10.2 and elderly 12.3) (Table 1).

## Discussion

Our study mainly tried to answer 2 questions, which were; (1) what is the relationship between BMI and BF %( measured by BIA)? And (2) what is the effect of age and gender in this relationship?, in a sub-population of South Asian adults. This was to determine the predictive value of BMI as a measure of BF% in adults, and to show any significance of age and sex in this prediction, which is not considered by WHO [1] at present when commenting obesity. Most researchers have tried to answer these questions in different populations and ethnic groups [3-7]. Some have also studied the predictive effect of racial difference in this (BMI- BF %) relationship [5,22]. We analysed data from a group of native Sri Lankan adults who are categorized as South Asians (who are similar to Asian Indians), who have a different body composition compared to Caucasians, Blacks and even Asian Mongolians [22-24]. We hope that answers to the above questions provided by this different ethnic group, would further add strength to the current pool of evidence regarding the relationship between BMI and BF%.

The use of different methods to estimate BF%; their validity, reliability have been discussed in variety of studies [25-27]. We used bioelectrical impedance analysis method (BIA) to estimate the BF% of our subjects. The use of BIA as a safe, valid and feasible tool is been accepted [17,19] and the equipment we used (BC-418, Tanita Corp, Tokyo, Japan)has been validated in past population based studies in different ethnic groups [20,21].

Our study confirmed the significant positive relationship between BMI and BF% which was demonstrated in most of the former studies. This was observed in both males and females separately and in each age group (young, middle-age and elderly). An early study by P. Deurenberg [7] done in Caucasians, this interaction was significant; whereas Jackson et al. [5] who compared Caucasians with Blacks reported the same. Rush et al. [22] who studied European, Maori, Pacific Islanders and Asian Indian adults also confirmed the significant positive relationship in BMI-BF% in all these races. More recent large study by S. Meeuwsen [9] using UK adults has shown that the association is not especially good. This is particularly so when BMI is less than 25 kg/m^2^, particularly in men. BMI values of most of our participants were between 20–30 kg/m^2^, whereas the BMI range varied among other studies. The reasons for these discrepancies observed in these cross sectional studies, said to be unknown and assumed to be due to, the use of different body composition methodology as well as biological differences in the characteristics of the study populations [9].

This study also tried to answer a controversial issue of linearity/curvilinearity of the studied relationship (BMI–BF %). Some former studies have shown the relationship as linear [6] and some as curvilinear/ quadratic [8,9]. Curvilinearity was mostly observed when the subjects of the samples had higher BMI values (35 kg/m^2^ or more) [8,9]. Study by Rush et al. described the relationship to be curvilinear, in Europeans (mean BMI 25 · 8 kg/m^2^, range 20 · 0–36 · 2), in Maori (30 · 4 kg/m^2^ / 18 · 9–43 · 5), in Pacific Island (31 · 3 kg/m^2^ / 17 · 8–42 · 2) and in Asian Indians (26 · 1 kg/m^2^ /16 · 8–40 · 2). But our total sample had 94% of the subjects having BMI values <30 kg/m^2^ (mean 23.8 kg/m^2^, range 14.8 - 41.1 kg/m^2^) and still showed a curvilinear relationship. Females who had a better fit of the curvilinear curve had 8.8% of the population having BMI values >30 kg/m^2^. Males who had a lesser fit of the curve had 4.8%. This shows the effect of curvilinearity with higher BMI values, which was discussed by other authors. But in this Sri Lankan population curvilinearity became significant in BMI values <35 kg/m^2^ (even less than 30 kg/m^2^).

Multiple regression analysis conducted showed a significant effect of age and gender in the studied relationship (BMI- BF %) with more effect from gender (Beta(β) =0.586, p < 0.000). This effect of gender in the relationship is well documented in past studies [5-7,14,28]. The usual pattern in most populations was that, BF% is greater in women than in men [9], which we also observed in the full range of BMI values (p < 0.000). The effect of age was not studied previously as much as the effect of gender [5]. Our results confirmed the significant effect of age in the relationship which was consistent with the studies which did the same [5-7,9,22]. So we support the existing body of evidence that, when describing BMI values for prediction of BF%, (or overweight/obesity) the effect of gender and age to be considered, clinically and in public health interventions.

When considering the individual effect of age on BF% (age-BF %), we observed when subjects got older there was a linear increase of BF% in both sexes. But BMI increased with age (age-BMI) in a curvilinear manner, showing a reduction in BMI in elderly. So elderly subjects reduced BMI and increased BF% at the same time. This may be due to sarcopenia, which is explained by progressive loss of muscle mass with age and accumulation of body fat [29]. These changes are attributed to physical inactivity, motor-unit remodeling, decreased hormone levels, and decreased protein synthesis which occur with aging [29,30]. Sri Lankans at the moment one of the fastest aging populations in the region [31] will need further research determining the effects of sarcopenia; and interventions on reversing sarcopenia and associated cardiovascular risk [32]. The mean difference in BF% between males and females also increased with age, where females gained more fat than males when they became old. In this context, some of the interventions might have to be directed more towards females.

This study had several limitations. The sample was taken from a health conscious group of adults who attended a medical exhibition and we can’t generalize these data to all Sri Lankans. We were not able to control some of the BIA measurement prerequisites as we relied on information given by the participants (E.g. Measurements should be taken patient fasting for 3 hours/not having vigorous activity for past 12 hours). But the results of the study were comparable to other studies done in more controlled subject samples. Yet another factor is the accuracy of bioelectrical impedance technique when compared with reference body composition measurement techniques (hydrodensitometry, water dilution technique) or multicomponent models. However, in epidemiological studies some degree of accuracy is sacrificed for simplicity, acceptability and rapid data acquisition [9].

## Conclusions

Our results demonstrate that Body Mass Index (BMI) strongly correlates with body fat percentage (BF %) estimated by bioelectrical impedance, in this group of South Asian adults from Sri Lanka. This relationship was curvilinear in nature even at BMI values <30 kg/m^2^. It was significantly influenced by age and gender of the individual where gender affected most. Therefore our findings support the importance of taking age and gender in to consideration when using BMI to predict body fat percentage/obesity, in a population.

## Abbreviations

BMI: Body mass index; BF%: Body fat percentage; BIA: Bioelectrical impedance analysis; WHO: World Health Organisation; UK: United Kingdom; SPSS: Statistical package for the social sciences.

## Competing interests

The authors declare that they have no competing interests.

## Authors’ contributions

CR, PG participated in the design of the study, data collection, examining the participants, performed the statistical analysis and drafted the manuscript. PK, NA participated in its design and helped to draft the manuscript. ST, PT data collection, examining the participants and helped to draft the manuscript. All authors read and approved the final manuscript.

## Pre-publication history

The pre-publication history for this paper can be accessed here:

http://www.biomedcentral.com/1471-2458/13/797/prepub
